# Association between the stress hyperglycemia ratio and all-cause mortality in critically ill patients with T2DM: a retrospective study

**DOI:** 10.3389/fendo.2025.1487496

**Published:** 2025-03-18

**Authors:** Yuanyuan Rui, Bing Wu, Changbao Huang, Qian Li

**Affiliations:** ^1^ Department of Emergency, the First Affiliated Hospital of Wannan Medical College, Wuhu, Anhui, China; ^2^ Department of Emergency, the Second People’s Hospital of Lu’an City, Lu’an, Anhui, China

**Keywords:** SHR, all-cause mortality, MIMIC-IV, T2DM, critically ill patients

## Abstract

**Background:**

Previous studies have shown a significant correlation between the stress-hyperglycemia ratio (SHR) and mortality. However, it is unknown whether the SHR has the same predictive value in severely ill patients. The main purpose of this research was to investigate the association between the SHR and all-cause mortality in critically ill patients with T2DM.

**Methods:**

The data used in this study were derived from the Medical Information Mart for Intensive Care (MIMIC-IV) database. The primary outcome was 180-day mortality and the secondary outcomes were 28-day, 90-day and 365-day mortality. The main analytical methods included: Kaplan-Meier survival analysis, the COX proportional hazards model and restricted cubic splines.

**Results:**

A total of 993 patients were included. The 28-day, 90-day, 180-day, and 365-day mortalities reached 10.4%, 14.4%, 16.7% and 19.0%, respectively. Multivariate Cox proportional hazards analysis revealed that the elevated SHR was significantly related to 28-day, 90-day and 180-day all-cause mortality even after cofounder adjustment. Restricted cubic spline analysis revealed a nonlinear association between the SHR and the risk of 28-day (p for nonlinear=0.014), 90-day (p for nonlinear=0.007), 180-day (p for nonlinear=0.001) and 365-day (p for nonlinear=0.003) all-cause mortality.

**Conclusion:**

SHR is significantly associated with 28-day, 90-day and 180-day all-cause mortality in critically ill patients with T2DM. This may help us identify patients at higher risk of death early.

## Introduction

With the development of medical technology, the monitoring and treatment measures for critically ill patients are becoming increasingly abundant, providing patients with meticulous medical care. This trend is placing an increasing burden on the global health economy. Notably, nearly 12% of global health spending in 2015 was spent on tackling T2DM and its associated complications (1). Patients with T2DM are more likely to develop complications and even die during hospitalization (2). Therefore, for patients with T2DM, early screening, early diagnosis and early intervention are essential to improve their prognosis.

SHR, an abbreviation for the stress-hyperglycemia ratio, is a new clinical marker that has been proposed in recent years. It is calculated using the formula: SHR = (admission blood glucose in mg/dL)/(28.7 × HbA1c (%) - 46.7). This value is calculated on the basis of blood glucose and HbA1c measured at admission (3). Previous studies have demonstrated a nonlinear association between the SHR and adverse endpoints in severely ill patients (4–6). However, in severely ill people with T2DM, whether the SHR has the same predictive value is unknown. Therefore, the main purpose of this paper is to study the above relationship.

## Materials

### Study population

**Figure 1:**
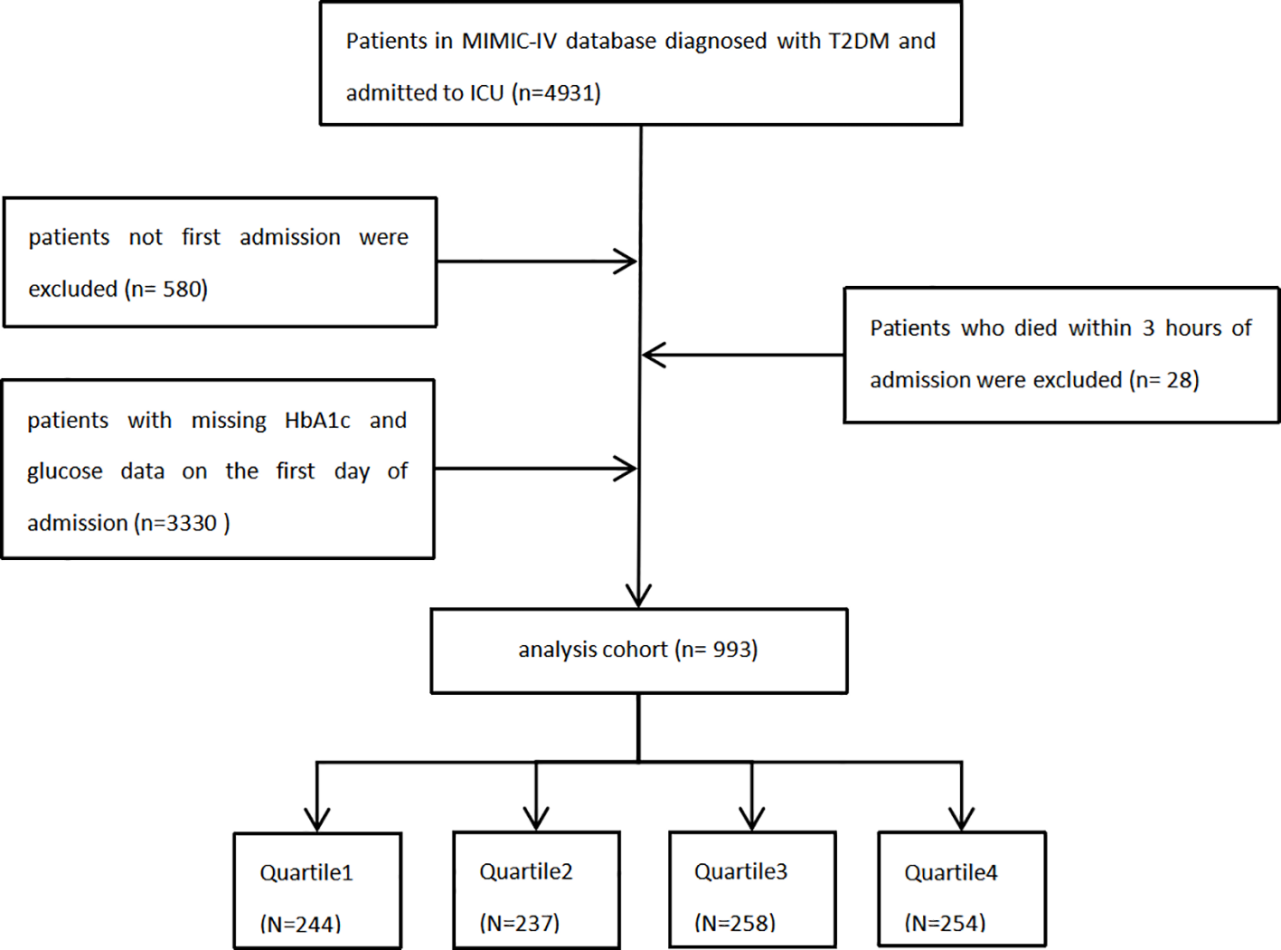
Flow chart of study participants.

This study was a retrospective analysis utilizing data from the MIMIC-IV database (version 2.2), whose full name is the Medical Information Mart for Intensive Care Database (7). The author (Yuan Rui) followed the requirements for database access and was responsible for the extraction of the data. We included patients diagnosed with T2DM who were admitted to the (ICU) during their hospitalization, based on data extracted from the MIMIC-IV database. The following groups of people were excluded (1): patients under 18 years old at the time of their initial admission (2); patients with multiple ICU admissions, from whom only data from the first admission were collected (3); patients who passed away within 3 hours of admission; and (4) patients lacking adequate data (ABG and HbA1c) on the first day of admission. Finally, 993 participants were included in this study, which categorized them into four groups according to the quartiles of the SHR index (**Figure 1**).

### Data collection

The software PostgreSQL (version 13.7.2) was utilized to retrieve information. The extracted data can be divided into the following categories: Demographics, past medical history, Laboratory parameters and Severity of illness scores. The calculation of the stress-hyperglycemia ratio (SHR) is as follows (3):


SHR=(admission blood glucose in mg/dl)/(28.7×HbA1c (%)-46.7)

All variables were obtained from the data collected within 24 hours of admission.

To prevent potential bias, variables with over 20% missing values are eliminated. For those with less than 20% missing values, the “missForest” package in R Studio was used to impute the data.

### Clinical outcomes

The main outcome of this study was all-cause mortality at 180 days. The secondary outcomes included all-cause mortality at 28 days, 90 days, and 365 days.

### Statistical analysis

Continuous variables are categorized into normally distributed and nonnormally distributed data. Normally distributed data are presented as means ± standard deviations, and analysis of variance (ANOVA) is employed to assess differences between groups. In contrast, nonnormally distributed data are represented as medians with interquartile ranges (IQRs), with the Kruskal-Wallis test used for group comparisons. Categorical variables are expressed as percentages, and chi-square tests are utilized to evaluate differences between groups. The K-M survival analysis was employed to assess the occurrence of endpoint events in each group, and the log-rank test was utilized to compare differences between groups.

We divided patients into four groups: quartile 1 with SHR ≤ 0.81, quartile 2 with 0.81<SHR ≤ 1.00, quartile 3 with 1.00<SHR ≤ 1.29, quartile 4 with SHR>1.29. First, we identified variables that were associated with outcomes via univariate Cox regression analysis. We set up three models for multivariate Cox regression analysis. We then employed the Cox proportional hazards model to examine the relationships between the SHR and the outcomes. The SHR was assessed using both nominal and continuous variables. The P values for trends were determined based on quartile levels.

Additionally, RCS regression model analysis was used to explore nonlinear correlation between the SHR and endpoints. To further investigate the effects of risk factors on the associations between the SHR and outcomes, we performed subgroup analyses. P-values for interactions are shown on the graphs.

A two-tailed p-value below 0.05 was deemed statistically significant. All the statistical analysis were conducted using R software (version 4.4.0) and StataMP 16.0 (Stata Corp., College Station, TX, USA).

## Results

In total, 993 participants were included in our study. Of these, 629 participants were male, with a mean age of 68.1 years. The overall mean of SHR is 1.1. The mean length of hospital stay and mean ICU stay were 7.8 days and 2.1 days, respectively. The mortality rates at 28 days, 90 days, 180 days, and 365 days were 10.4%, 14.4%, 16.7%, and 19.0%, respectively (**Table 1**).

**Table 1:** Characteristics and outcomes of patients grouped by SHR quartiles^a^.

	Total (n=993)	Q1 (n=244)	Q2 (n=237)	Q3 (n=258)	Q4 (n=254)	p
SHR	1.1 ± 0.4	0.6 ± 0.1	0.8 ± 0.0	1.1 ± 0.0	1.7 ± 0.5	<0.001
Male [n (%)]	629 (63.3)	147 (60.2)	161 (67.9)	159 (61.6)	162 (63.7)	0.320
Age (years)	68.1 ± 12.2	65.2 ± 12.5	69.2 ± 12.0	68.8 ± 12.0	69.0 ± 12.0	<0.001
Weight (kg)	89.9 ± 24.8	89.6 ± 27.0	89.2 ± 22.5	91.1 ± 25.4	88.9 ± 24.1	0.502
Race [n (%)]						0.935
Black	109 (10.9)	32 (13.1)	25 (10.5)	26 (10.0)	26 (10.2)	
White	516 (51.9)	121 (49.5)	122 (51.4)	138 (53.4)	135 (53.1)	
Hispanic	45 (4.5)	9 (3.6)	14 (5.9)	10 (3.8)	12 (4.7)	
Other	323 (32.5)	82 (33.6)	76 (32.0)	84 (32.5)	81 (31.8)	
Laboratory tests						
Serum sodium (mEq/L)	138.5 ± 4.7	139.3 ± 3.7	138.7 ± 4.4	138.8 ± 3.7	137.4 ± 6.3	<0.001
Serum potassium (mEq/L)	4.2 ± 0.7	4.2 ± 0.7	4.2 ± 0.5	4.1 ± 0.4	4.5 ± 1.0	<0.001
Serum chloride (mEq/L)	101.4 ± 5.5	102.4 ± 4.6	101.3 ± 4.8	101.6 ± 4.6	100.2 ± 7.2	<0.001
Serum creatinine (mg/dL)	1.3 ± 1.3	1.2 ± 1.1	1.2 ± 1.3	1.2 ± 1.0	1.6 ± 1.6	<0.001
Hemoglobin (g/dL)	12.1 ± 2.1	12.0 ± 2.1	12.4 ± 2.1	12.2 ± 1.8	11.9 ± 2.2	0.054
WBC (K/uL)	10.3 ± 5.2	9.5 ± 4.2	9.8 ± 6.3	10.0 ± 4.2	11.8 ± 5.4	<0.001
Platelet (K/uL)	213.5 ± 74.1	213.8 ± 76.7	209.8 ± 67.6	214.3 ± 71.3	215.9 ± 80.2	0.827
BUN (mg/dL)	23.6 ± 17.0	21.8 ± 14.6	21.2 ± 14.3	21.0 ± 11.4	30.5 ± 23.4	<0.001
Bicarbonate (mEq/L)	23.2 ± 4.3	23.4 ± 4.2	24.0 ± 3.9	23.5 ± 3.8	21.8 ± 5.0	<0.001
Anion gap (mEq/L)	15.6 ± 4.0	14.8 ± 3.6	14.9 ± 3.1	15.2 ± 3.5	17.2 ± 5.0	<0.001
HbA1c (%)	7.7 ± 2.0	8.4 ± 2.4	7.5 ± 1.9	7.4 ± 1.7	7.5 ± 2.0	<0.001
BG (mg/dL)	191.0 ± 104.1	124.0 ± 44.2	153.4 ± 51.6	187.7 ± 57.6	293.7 ± 136.1	<0.001
Scores						
sofa	1 (0, 3)	1 (0, 3)	1 (0, 3)	1 (0, 3)	2 (0, 4)	0.646
GCS	33 (26, 40)	32 (24, 40)	32 (25, 39)	33 (26, 39)	36 (27, 44)	<0.001
SAPSII	15 (15, 15)	15 (15, 15)	15 (15, 15)	15 (15, 15)	15 (15, 15)	0.464
Commorbidities						
MI	287 (28.9)	68 (27.8)	72 (30.3)	66 (25.5)	81 (31.8)	0.413
HBP	491 (49.4)	126 (51.6)	123 (51.8)	138 (53.4)	104 (40.9)	0.018
CKD	242 (24.3)	58 (5.8)	48 (20.2)	58 (22.4)	78 (30.7)	0.041
HF	292 (29.4)	63 (6.3)	70 (29.5)	67 (25.9)	92 (36.2)	0.034
Sepsis	407 (40.9)	88 (36.0)	85 (35.8)	104 (40.3)	130 (51.1)	0.001
AF	340 (34.2)	70 (28.6)	81 (34.1)	93 (36.0)	96 (37.7)	0.162
COPD	109 (10.9)	22 (9.0)	21 (8.8)	25 (9.6)	41 (16.1)	0.024
Outcomes						
Los_icu	2.1 (1.3,4.2)	1.9 (1.2,3.7)	2.1 (1.2,4.1)	2.2 (1.3,4.5)	2.4 (1.3,4.9)	0.027
Los_hos	7.8 (5.0,11.9)	7.9 (5.1,12.3)	7.6 (4.9,11.9)	7.9 (5.2,11.7)	7.8 (4.6,12.6)	0.716
Death28	104 (10.4)	16 (6.5)	22 (9.2)	21 (8.1)	45 (17.7)	<0.001
Death90	143 (14.4)	26 (10.6)	29 (12.2)	29 (11.2)	59 (23.2)	<0.001
Death180	166 (16.7)	33 (13.5)	35 (14.7)	32 (12.4)	66 (25.9)	<0.001
Death365	189 (19.0)	43 (17.6)	43 (18.1)	33 (12.7)	70 (27.5)	<0.001

a SHR quartiles: Q1 (≤0.81),Q2 (0.81<SHR ≤ 1.00),Q3 (1.00<SHR ≤ 1.29),Q4 (SHR>1.29).

SHR, stress hyperglycemia ratio; WBC, white blood cell; BUN, blood urea nitrogen; BG, blood glucose; SOFA, sequential organ failure assessment; GCS, Glasgow coma scale; SAPS II, simplified acute physiology score II; MI, myocardial infarction; HBP, high blood pressure; CKD, chronic kidney disease; HF, heart failure; AF, atrial fibrillation; COPD, chronic obstructive pulmonary disorder; Los, length of stay.

### Baseline characteristics

As shown in **Table 1**, we categorized participants into four groups according to the SHR measured at admission, and presented demographic characteristics, laboratory parameters, past medical history, scores, length of hospital stay, and survival outcomes in each of the four groups. The SHR levels of the four groups were as follows: quartile 1 (SHR ≤ 0.81), quartile 2 (0.81<SHR ≤ 1.00, quartile 3 (1.00<SHR ≤ 1.29), and quartile 4 (SHR>1.29). The mean SHR values for each group were: 0.6 ± 0.1, 0.8 ± 0.0, 1.1 ± 0.0, and 1.7 ± 0.5. Participants in the higher quartile of SHR index generally had higher age, serum sodium, serum potassium, serum chloride, serum creatinine, WBC, blood glucose, HbA1c, BUN, bicarbonate, anion gap, higher GCS scores and higher prevalence of hypertension, CKD, heart failure, sepsis and COPD compared to the lower quartile. Patients with higher SHR levels had longer hospital stays and ICU stays, and their 28-day, 90-day, 180-day, and 365-day mortality rates were higher than those with lower SHR levels.

### Clinical outcomes

**Figure 2:**
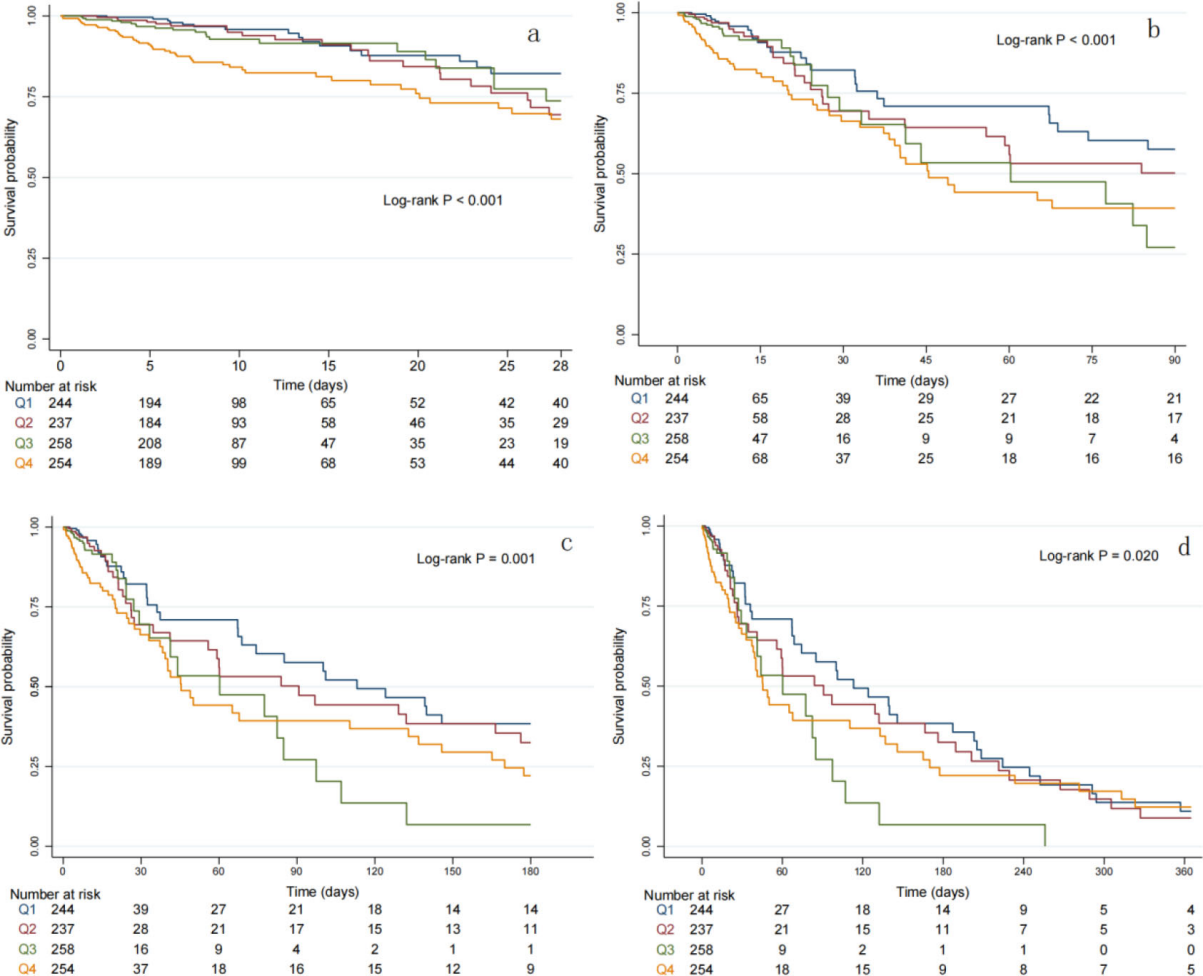
Kaplan-Meier survival analysis curves for all-cause mortality. K-M curves showing the survival probability of all-cause mortality according to groups at 28 days **(a)**, 90 days **(b)**, 180 days **(c)** and 365 days **(d)**.

**Figure 3:**
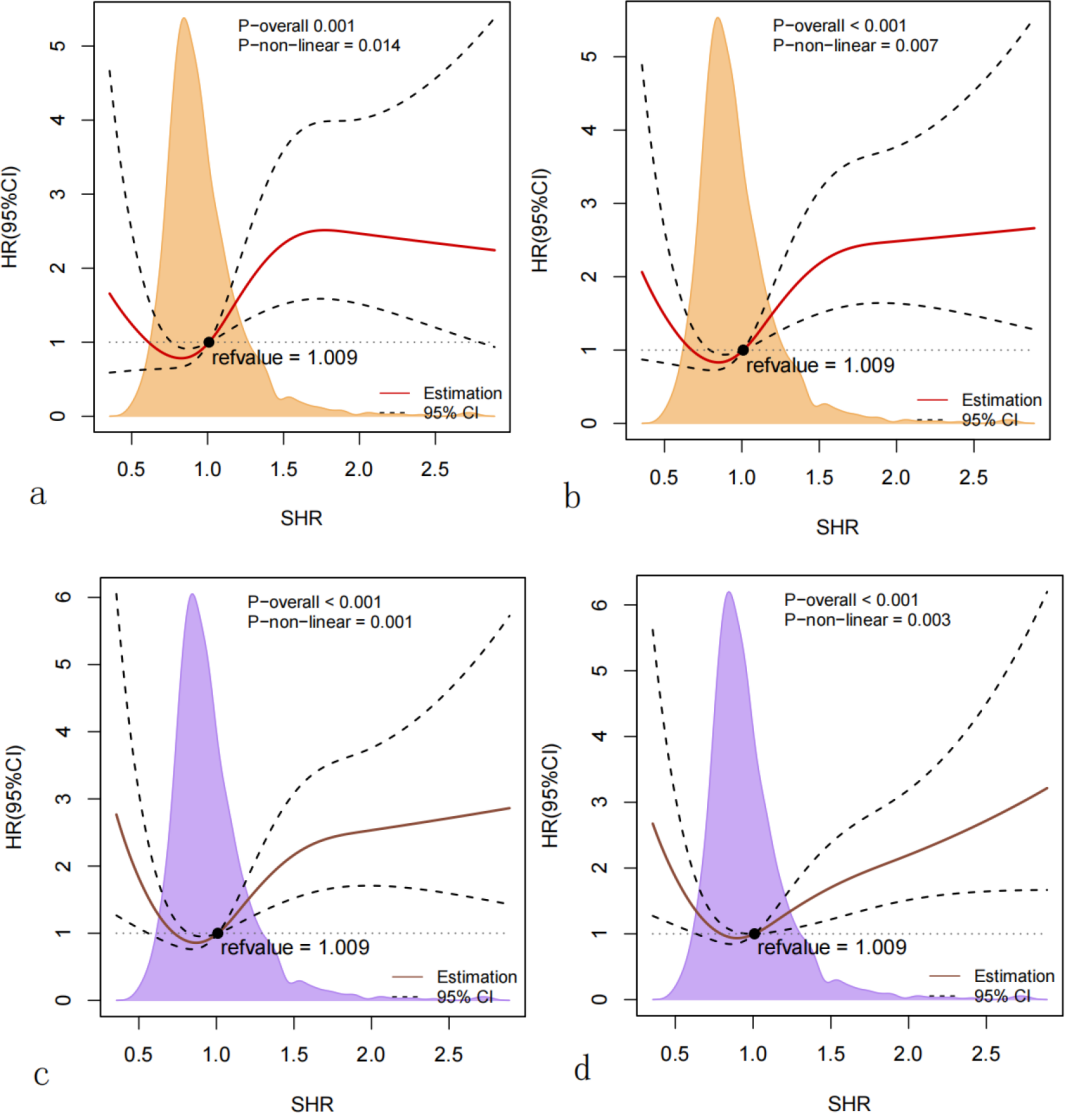
**(a)** Restricted cubic spline for 28-day mortality; **(b)** restricted cubic spline for 90-day mortality; **(c)** restricted cubic spline for 180-day mortality; **(d)** restricted cubic spline for 365-day mortality. HR, hazard ratio; CI, confidence interval; SHR, stress hyperglycemia ratio.

As is shown in **Figure 2**, Kaplan-Meier survival analysis curves were utilized to examine the occurrence of the primary outcome and secondary outcomes across the four groups categorized by the quartiles of the SHR index. Patients with a higher SHR index had a higher risk of 28-day, 90-day, 180-day and 365-day mortality, and the differences were significant (log-rank p<0.001,<0.001, 0.001, 0.020 respectively). Univariate Cox regression analysis was used to explore the relationships between variables and 180-day mortality in critically ill patients with T2DM. Moreover, we also included a subset of variables that may influence the endpoints based on clinical experience. Ultimately, we identified gender, age, hemoglobin, sepsis, MI, HBP, HF and SAPSII as influential factors. The results demonstrated that when the SHR was a continuous variable, it was a significant risk factor in Model 1, but in Model 2 and Model 3, there was no statistically significant difference between SHR and 180-day mortality. When the SHR was treated as a nominal variable, patients in the highest quartile of the SHR showed a significant association with an increased risk of 180-day mortality across all three established Cox proportional hazards models: Model 1[HR, 2.16 (1.42-3.28) P<0.001], Model 2[HR, 1.70 (1.24-2.23) P=0.001], Model 3[HR, 1.55 (1.11-2.14) P=0.008]. When we took 28-day, 90-day, 365-day mortality as the endpoints, we could get similar outcomes (**Tables 2**, **3**). At last, the RCS regression model was used to explore the nonlinear relationships between the SHR and 28-day, 90-day, 180-day and 365-day mortality ((P for non-linearity=0.014, 0.007, 0.001, 0.003, respectively) (**Figure 3**).

**Table 2:** Cox proportional hazard ratios (HRs) for 28-day and 90-day all-cause mortality.

Categories	Model 1			Model 2			Model 3		
	HR (95%CI)	P-value	P for trend	HR (95%CI)	P-value	P for trend	HR (95%CI)	P-value	P for trend
28-days mortality									
SHR as continuous	1.48 (1.11-1.96)	0.007		1.39 (1.03-1.86)	0.027		1.20 (0.89-1.63)	0.219	
Quartile ^a^			<0.001			0.001			0.003
Q1 (n=244)	Ref.			Ref.			Ref.		
Q2 (n=237)	1.52 (0.79-2.89)	0.201		1.21 (0.71-2.06)	0.475		1.29 (0.75-2.20)	0.343	
Q3 (n=258)	1.49 (0.77-2.86)	0.229		1.33 (0.69-2.56)	0.386		1.41 (0.73-2.73)	0.297	
Q4 (n=254)	2.79 (1.58-4.95)	<0.001		2.01 (1.36-2.97)	<0.001		1.81 (1.21-2.71)	0.003	
90-days mortality									
SHR as continuous	1.35 (1.07-1.71)	0.011		1.29 (1.01-1.65)	0.035		1.11 (0.87-1.42)	0.394	
Quartile ^a^			<0.001			<0.001			0.002
Q1 (n=244)	Ref.			Ref.			Ref.		
Q2 (n=237)	1.28 (0.75-2.18)	0.354		1.21 (0.71-2.07)	0.464		1.34 (0.78-2.29)	0.276	
Q3 (n=258)	1.58 (0.93-2.71)	0.089		1.30 (0.83-2.06)	0.246		1.40 (0.88-2.22)	0.148	
Q4 (n=254)	2.39 (1.50-3.79)	<0.001		1.84 (1.31-2.57)	<0.001		1.61 (1.14-2.28)	0.007	

Model 1: unadjusted.

Model 2: adjusted for age, male.

Model 3: adjusted for age, male, hemoglobin, sepsis, MI, hypertension, heart failure, SAPS II.

a SHR quartiles: Q1 (≤0.81),Q2 (0.81<SHR ≤ 1.00),Q3 (1.00<SHR ≤ 1.29),Q4 (SHR>1.29).

**Table 3:** Cox proportional hazard ratios (HRs) for 180-day and 365-day all-cause mortality.

Categories	Model 1			Model 2			Model 3		
	HR (95%CI)	P-value	P for trend	HR (95%CI)	P-value	P for trend	HR (95%CI)	P-value	P for trend
180-days mortality									
SHR as continuous	1.26 (1.01-1.56)	0.033		1.21 (0.97-1.51)	0.084		1.07 (0.86-1.34)	0.519	
Quartile ^a^			<0.001			<0.001			0.002
Q1 (n=244)	Ref.			Ref.			Ref.		
Q2 (n=237)	1.26 (1.01-1.56)	0.381		1.20 (0.74-1.93)	0.452		1.28 (0.78-2.07)	0.316	
Q3 (n=258)	1.63 (0.99-2.67)	0.053		1.38 (0.90-2.13)	0.137		1.43 (0.93-2.21)	0.100	
Q4 (n=254)	2.16 (1.42-3.28)	<0.001		1.70 (1.24-2.32)	0.001		1.55 (1.11-2.14)	0.008	
365-days mortality									
SHR as continuous	1.19 (0.97-1.45)	0.092		1.15 (0.93-1.42)	0.173		1.03 (0.83-1.26)	0.772	
Quartile ^a^			0.002			0.005			0.014
Q1 (n=244)	Ref.			Ref.			Ref.		
Q2 (n=237)	1.17 (0.76-1.79)	0.457		1.17 (0.76-1.79)	0.464		1.28 (0.83-1.97)	0.263	
Q3 (n=258)	1.48 (0.92-2.36)	0.098		1.30 (0.85-1.97)	0.214		1.35 (0.89-2.06)	0.154	
Q4 (n=254)	1.75 (1.19-2.56)	0.004		1.45 (1.08-1.96)	0.013		1.31 (0.97-1.79)	0.077	

Model 1: unadjusted.

Model 2: adjusted for age, male.

Model 3: adjusted for age, male, hemoglobin, sepsis, MI, hypertension, heart failure, SAPS II.

a SHR quartiles: Q1 (≤0.81),Q2 (0.81<SHR ≤ 1.00),Q3 (1.00<SHR ≤ 1.29),Q4 (SHR>1.29).

### Subgroup analysis

**Figure 4:**
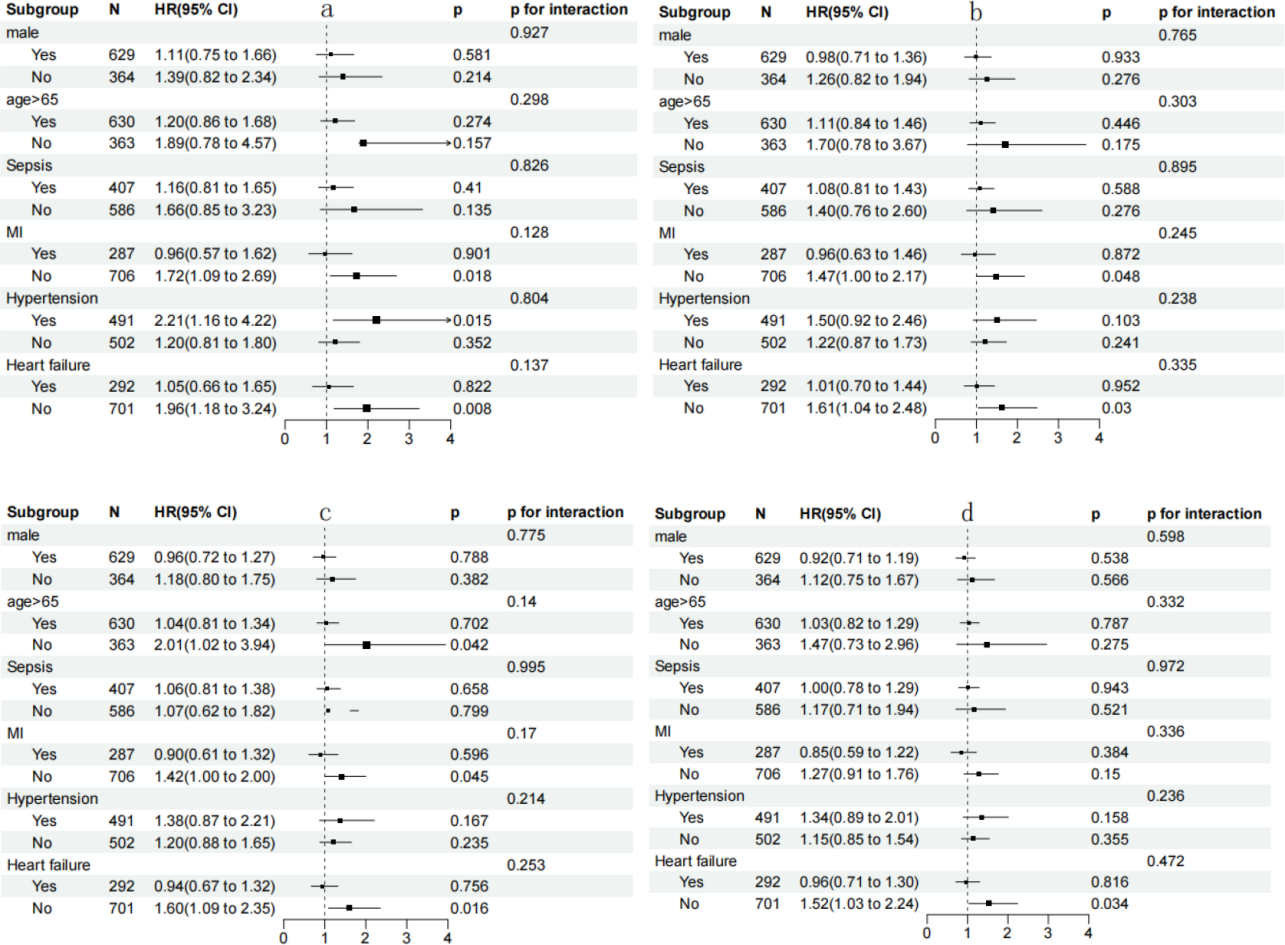
**(a)** Forest plots of hazard ratios for 28-day mortality; **(b)** Forest plots of hazard ratios for 90-day mortality; **(c)** Forest plots of hazard ratios for 180-day mortality; **(d)** Forest plots of hazard ratios for 365-day mortality. HR, hazard ratio; CI, confidence interval; MI, myocardial infarction.

The SHR index was found to be significantly linked to an increased risk of 180-day mortality in subgroups of T2DM patients aged 65 years or younger [HR (95% CI) 1.481 (1.126-1.950)], those without a history of myocardial infarction [HR (95% CI) 1.214 (1.000-1.474)], and those without heart failure. Additionally, in the stratified analyses for 28-day and 90-day mortality, the SHR index also showed a significant correlation with an increased probability of mortality in subgroups of patients without myocardial infarction and those without heart failure (**Figure 4**).

## Discussion

Our findings revealed that an increased SHR index was linked to increased adverse outcomes at 28 days, 90 days, and 180 days in these individuals. Notably, after accounting for confounding factors, there was still a significant association between the SHR and outcomes. We also studied the nonlinear correlation between the SHR and prognosis. It was found that there was a U-shaped correlation between the two. This correlation suggests that both an increase and a decrease in the level of the SHR leads to an increase in mortality. Thus, this finding is significant for clinicians to predict the outcomes of patients in clinical practice.

Critically ill patients hospitalized in the ICU often develop SHR. Research has shown that nearly half of patients in the ICU have develop stress hyperglycemia (8). The basic mechanism is mainly related to the hypothalamic-pituitary-adrenal (HPA) axis and the sympathetic-adrenal system (9). This stress response is even more intense because patients in the ICU are critically ill. To date, a number of studies have found the predictive impact of the SHR on mortality. Jin Liu et al. revealed a nonlinear correlation of the SHR and adverse endpoints in individuals with MI in both American and Chinese cohorts (6). Another study of acute coronary syndromes had similar findings (10). Fengjuan Yan and his colleagues found that the correlation between SHR and the adverse endpoints was U-shaped in individuals with sepsis (5).

SHR is an important measure that helps mitigate the impact of prolonged chronic glycemic factors on stress hyperglycemia levels, thus providing a precise representation of the body’s physiological stress response. In the available literature, studies on the association between the SHR and mortality are still relatively scarce. Recently, Wei Xu conducted a study that found an elevated SHR to be an risk factor for mortality in the ICU among a cohort of 8,196 patients with coronary artery disease (11). Interestingly, they also performed restricted cubic spline analysis and found that in-hospital mortality increased linearly with the gradual rise of SHR. In contrast, Lei Ding and colleagues discovered a nearly U-shaped correlation between SHR and all-cause mortality in individuals with prediabetes (12). In line with these variations, our research also demonstrated a U-shaped correlation between the SHR and the outcomes in individuals with T2DM.

The U-shaped relationship indicates that both an increase and a decrease in the SHR are detrimental to prognosis. In other words, a mild-to-moderate SHR is protective of the patient prognosis. This finding coincides with those of several previous studies (9, 13). The exact mechanisms behind the U-shaped correlation are still unclear. Previous studies might provide insight into the underlying mechanisms involved. Stress hyperglycemia is believed to be a physiological reaction aimed at reestablishing homeostasis during times of significant stress. The body’s metabolic processes rely on a prompt supply of glucose. This concept is underscored by studies involving animal models of hemorrhagic shock, which demonstrated that administering a hypertonic glucose solution led to enhanced cardiac output, elevated blood pressure, and improved survival rates (14). A basic research on myocardial infarction found that hyperglycemia decreases the release of proinflammatory factors and activation of apoptosis, and increases the release of cell growth factors. As a result, there was a decrease in myocardial infarction size and myocardial fibrosis, and improvements in cardiac systolic function (15). Therefore, moderate hyperglycemia has a very important protective effects on the body’s metabolism and inflammatory response. These basic studies are consistent with the conclusions of clinical studies. The SHR is capable of forecasting negative outcomes in patients with heart failure (16–19), stroke (20, 21) and pneumonia (22).

Patients with low SHR tend to have hypoglycemia, while patients with high SHR tend to have stress hyperglycemia, both of which can cause disruption to the body’s internal environment. Patients who develop hypoglycemia during hospitalization are more likely to develop conditions such as heart failure and AKI. They also have a higher in-hospital mortality rate (23). Some basic experiments have found that hypoglycemia can activate platelets (24) and increase fatty acid concentrations (25), thereby inducing cardiovascular and cerebrovascular complications. In addition, hypoglycemia activates the body’s sympathetic-adrenal system, causing an increase in heart rate and increased cardiac output (26). Sometimes, hypoglycemia directly affects the heart’s electrical activity, causing arrhythmia (27). High SHR levels tend to indicate stress hyperglycemia. Hyperglycemia may trigger inflammation (28), endothelial cell dysfunction (29), and oxidative stress (30), resulting in dysfunction of the microcirculation. For patients with severe disease, these pathophysiological processes are detrimental to the outcome of the disease, especially for those who are older or have more underlying diseases.

High SHR represents stress-induced hyperglycemia, which has been demonstrated in multiple studies to be associated with poor prognosis (31). Under stress conditions, the body elevates blood glucose levels to provide additional energy in response to acute injury or severe infection. However, this short-term adaptive response may lead to persistent hyperglycemia in critically ill patients, triggering oxidative stress, exacerbating inflammatory responses, impairing immune function, and causing endothelial damage. These detrimental effects increase the risk of infection, organ dysfunction, and mortality. Although hypoglycemia also poses certain risks to the body, such as arrhythmias, neurological impairment, and increased mortality, the impact of high SHR on organ function through inflammation, oxidative stress, and metabolic dysregulation is more pronounced in critically ill patients. Moreover, the incidence of hypoglycemia in ICU and critically ill patients is relatively low and can typically be managed with timely glucose supplementation. In contrast, high SHR is often accompanied by insulin resistance and exacerbated inflammation, making treatment more challenging and leading to more significant long-term consequences. Therefore, in critically ill patients, high SHR may present greater clinical harm than low SHR.

In conclusion, previous research have established a connection between stress hyperglycemia and clinical outcomes. Alongside our findings, this highlights the critical need to maintain an optimal SHR, as substantial deviations—whether too high or too low—can result in adverse health consequences.

Inevitably, this study has several shortcomings. First, due to the limitations of the database, we were not able to include some variables outside the database, which may had some impact on outcomes in actual clinical practice. Secondly, since this is an observational study, a definitive causal relationship between the variable and the outcome cannot be determined. This depends on future prospective multicenter studies to further investigate the association between the them. Third, we included only the initial SHR of patients within 24 hours of admission, and did not pay attention to the dynamics of the SHR, which may be more strongly related to patient outcomes.

## Conclusion

In this study, the SHR index proved to be an effective predictor of all-cause mortality risk in patients with T2DM. A U-shaped correlation was observed between the SHR and short-term and long-term outcomes. Testing for the SHR helps to make the right clinical decisions in the course of clinical practice. Whether there is a causal relationship between SHR and mortality requires further research.

## Data Availability

The original contributions presented in the study are included in the article/**Supplementary Material**. Further inquiries can be directed to the corresponding author.
