# Supplementary material for: Association between the stress hyperglycemia ratio and all-cause mortality in critically ill patients with T2DM: a retrospective study
Source: Front Endocrinol (Lausanne). 2025 Mar 18;16:1487496. doi: 10.3389/fendo.2025.1487496 (PMC11958164; doi:10.3389/fendo.2025.1487496)
Supplement: Supplementary file 1 [file Table1.docx]

**Table S1. univariate COX regression analysis of incidence of 180-day mortality**

| Categories | HR(95%CI) | p |
| --- | --- | --- |
| SHR  Male [n(%)] | 1.26(1.01-1.56)  0.63(0.47-0.86) | 0.033  0.004 |
| Age (years)  Weight(kg) | 1.02(1.00-1.03)  0.99(0.99-1.00) | 0.001  0.674 |
| Race [n(%)] | 0.90(0.77-1.06) | 0.237 |
| Serum sodium(mEq/L) | 0.98(0.95-1.01) | 0.266 |
| Serum potassium(mEq/L) | 1.12(0.94-1.34) | 0.180 |
| Serum chloride(mEq/L) | 0.98(0.96-1.01) | 0.294 |
| Serum creatinine(mg/dL) | 1.03(0.95-1.13) | 0.403 |
| Hemoglobin(g/dL) | 0.90(0.85-0.97) | 0.004 |
| WBC(K/uL) | 1.01(0.99-1.03) | 0.089 |
| Platelet(K/uL) | 1.00(0.99-1.00) | 0.239 |
| BUN(mg/dL) | 1.00(0.99-1.00) | 0.514 |
| Bicarbonate(mEq/L) | 0.96(0.93-1.00) | 0.063 |
| Anion gap(mEq/L) | 1.03(0.99-1.06) | 0.068 |
| SOFA | 1.05(0.98-1.12) | 0.122 |
| GCS  SAPSII | 0.96(0.91-1.02)  1.03(1.02-1.04) | 0.281  <0.001 |
| MI | 1.51(1.09-2.10) | 0.012 |
| HBP | 0.66(0.47-0.91) | 0.014 |
| CKD | 1.28(0.93-1.75) | 0.122 |
| HF | 1.74(1.28-2.36) | <0.001 |
| Sepsis  AF | 1.52(1.09-2.11)  1.16(0.85-1.59) | 0.012  0.319 |
| COPD | 1.29(0.88-1.89) | 0.190 |

Abbreviations: SHR, stress hyperglycemia ratio; WBC, white blood cell; BUN, blood urea nitrogen; BG, blood glucose; SOFA, sequential organ failure assessment; GCS, Glasgow coma scale; SAPS II, simplified acute physiology score II; MI, myocardial infarction; HBP, high blood pressure; CKD, chronic kidney disease; HF, heart failure; AF, atrial fibrillation; COPD, chronic obstructive pulmonary disorder
